# Analgesic and Anti-Inflammatory Effects of Perampanel in Acute and Chronic Pain Models in Mice: Interaction With the Cannabinergic System

**DOI:** 10.3389/fphar.2020.620221

**Published:** 2021-02-01

**Authors:** Carmen De Caro, Claudia Cristiano, Carmen Avagliano, Mariarosaria Cuozzo, Giovanna La Rana, Gabriella Aviello, Giovambattista De Sarro, Antonio Calignano, Emilio Russo, Roberto Russo

**Affiliations:** ^1^ Department of Health Sciences, School of Medicine, University of Catanzaro, Catanzaro, Italy; ^2^ Department of Pharmacy, University of Naples Federico II, Naples, Italy

**Keywords:** perampanel, AMPA receptor, pain, inflammation, CB1 receptor

## Abstract

Pain conditions, such as neuropathic pain (NP) and persistent inflammatory pain are therapeutically difficult to manage. Previous studies have shown the involvement of glutamate receptor in pain modulation and in particular same of these showed the key role of the AMPA ionotropic glutamate receptor subtype. Antiseizure medications (ASMs) are often used to treat this symptom, however the effect of perampanel (PER), an ASM acting as selective, non-competitive inhibitor of the AMPA receptor on the management of pain has not well been investigated yet. Here we tested the potential analgesic and anti-inflammatory effects of PER, in acute and chronic pain models. PER was given orally either in acute (5 mg/kg) or repeated administration (3 mg/kg/d for 4 days). Pain response was assessed using models of nociceptive sensitivity, visceral and inflammatory pain, and mechanical allodynia and hyperalgesia induced by chronic constriction injury to the sciatic nerve. PER significantly reduced pain perception in all behavioral tests as well as CCI-induced mechanical allodynia and hyperalgesia in acute regimen (5 mg/kg). This effect was also observed after repeated treatment using the dose of 3 mg/kg/d. The antinociceptive, antiallodynic and antihyperalgesic effects of PER were attenuated when the CB_1_ antagonist AM251 (1 mg/kg/i.p.) was administered before PER treatment, suggesting the involvement of the cannabinergic system. Moreover, *Ex vivo* analyses showed that PER significantly increased CB_1_ receptor expression and reduced inflammatory cytokines (i.e. TNFα, IL-1β, and IL-6) in the spinal cord. In conclusion, these results extend our knowledge on PER antinociceptive and antiallodynic effects and support the involvement of cannabinergic system on its mode of action.

## Introduction

Neuropathic pain (NP) is a chronic pain condition characterized by different symptoms, including abnormal increase of sensitivity to innocuous stimuli (allodynia), and/or exacerbated sensitivity to noxious stimuli (hyperalgesia) (Jensen and Finnerup, 2014). NP is usually caused by one or multiple injuries at the central and/or peripheral nervous system as a result of a cascade of neurobiological processes. There are many medications available for pain sufferers, ranging from over-the-counter products to prescription medications including analgesics, antidepressants and antiseizure medications. Recently, antiseizure medications (ASMs) have shown to play an important role in the management of pain and especially in chronic NP (Sidhu and Sadhotra, 2016) and their use – also in combination with opioid analgesics - has been approved by the Food and Drug Administration (Chaparro et al., 2012). Antiepileptic drugs (AEDs) exert their antiseizure as well as analgesic effects by blocking sodium channels, increasing GABA-mediated inhibition, binding on specific subtype of calcium channels, and reducing glutamate release or blocking its ionotropic receptors (Bialer, 2012; Sidhu and Sadhotra, 2016; Tomić et al., 2018).

Alpha-amino-3-hydroxyl-5-methyl-4-isoxazole-propionate (AMPA) receptors mediate fast excitatory transmission and have a critical role in synaptic plasticity in the spinal cord (Latremoliere and Woolf, 2009). They are tetramers consisting of dimers of four different subunits, GluA1-4, all of which are expressed in the dorsal horn (DH) (Polgár et al., 2008). Activation of AMPA receptor triggers intracellular cation influx resulting in membrane depolarization, which in turn leads to N-methyl-D-aspartate (NMDA) activation, an increased influx of Ca^2+^ in the intracellular space (Hanada et al., 2011). High levels of intracellular Ca^2+^ activate signal transduction pathways responsible for the hyper-excitability of post-synaptic neurons (Douyard et al., 2007), thus AMPA receptor activation may be critical in the initiation of pathophysiological changes resulting in pain development.

It has been reported that upregulation of AMPA receptors in DH neurons causes central sensitization for a long period of time (Gwak et al., 2007). Moreover, peripheral inflammatory pain induces upregulation of Ca^2+^ permeable AMPA receptors at both synapses and the extra synaptic membranes of DH interneurons (Chizh et al., 2000; Adedoyin et al., 2010) the latter being linked to persistent pain. Some studies claimed a prominent role of AMPA receptor modulation in (Davidson et al., 1997; McRoberts et al., 2001). For instance, several studies imply a prominent role of AMPA receptors in the initiation of NP, as well as in different neurodegenerative conditions (Adedoyin et al., 2010) (Gwak et al., 2007). Although the analgesic effect of AMPA antagonists is supported by emerging preclinical evidence (Khan et al., 2019), their use needs to be further tested and their clinical potential remain to be investigated.

PER [2-(2-oxo-1-phenyl-5-pyridin-2-yl-1,2-dihydro- pyridin-3-yl) benzonitrile hydrate 4:3] is the first selective non-competitive AMPA receptor antagonist approved for the treatment of epilepsy (Hanada et al., 2011). It exerts a broad spectrum of antiseizure activity in preclinical models of either partial or generalized seizures (Hanada et al., 2011; Russo et al., 2012; Russmann et al., 2016; Citraro et al., 2017) and in patients with epilepsy (Di Bonaventura et al., 2017; Leo et al., 2018; Potschka and Trinka, 2019). To date, a few studies in rats have shown the effects of PER in attenuating pain in chronic constriction injury-induced (CCI) models of NP and the partial involvement of the opioid system on its mode of action (Khangura et al., 2017; Hara et al., 2020). In this study, we aimed at exploring the analgesic effects of both single and repeated PER administration in specific rodent models of acute and chronic pain and studied the potential involvement of the cannabinergic system in its modulation of neuroinflammation at spinal cord level.

## Materials and Methods

### Animals

Male CD1 mice (25–30 g) were purchased from Charles River Italy (Lecco, Italy) and housed in cages in a room kept at 22 ± 1°C with a 12:12 h light/dark cycle. The animals were acclimated to their environment for 1 week and had ad libitum access to standard rodent diet. Procedures involving animals and their care were conducted in conformity with international and national law and policies (EU Directive 2010/63/EU for animal experiments) and approved by the Institutional Committee on the Ethics of Animal Experiments (CSV) of the University of Naples “Federico II” and by the Ministero della Salute under protocol no.996/2016-PR. At the end of all procedures, animals were euthanized by CO_2_ overdose. As suggested by the animal welfare protocol, all efforts were made to minimize animal suffering and to use only the number of animals necessary to produce reliable scientific data.

### Drugs and Chemicals

PER (EISAI S.r.l., Milan, Italy) was dissolved in 10% PEG, 5% Tween 80 and 85% water and administered by gavage either at 5 mg/kg (0.3 mL/mouse) dose for the oral single acute treatment or at 3 mg/kg (0.3 mL/mouse) dose for repeated treatments (4 consecutive days, at 9–10 am). AM251 (Sigma-Aldrich, Italy) was dissolved in DMSO and injected intraperitoneally (i.p.) 1 h before PER administration at the dose of 1 mg/kg. Formalin (5% in saline), λ-carrageenan (1% in saline) and all other products used were purchased from Tocris or Sigma-Aldrich (Italy).

### Acute Pain Models

#### Tail Flick and Hot Plate tests

Each mouse was tested 1, 3, and 5 h after the administration of the acute dose of 5 mg/kg or after the last dose of 3 mg/kg/d repeated treatment, in both tail flick and hot plate tests. In the first test, tail-flick was evoked by a source of radiant heat, which was focused on the dorsal surface of the tail (de Novellis et al., 2012). The cut-off imposed was 15 s to prevent tissue damage. In the second test, mice were placed on a 55.5 ± 0.5°C hot plate apparatus to measure the latency (s) of either first hind paw pain response or jumping off the plate. The cut-off imposed was 60s to avoid tissue damage. Control mice received vehicle only.

#### Acetic Acid Evoked Writhing

Mice were placed separately into cages and allowed to acclimate for at least 10 min and then visceral pain was induced by i.p., injection (0.5 mL/mouse) of 0.6% acetic acid. In both acute and repeated drug administration, tests started 1 h after PER treatment (after the fourth dose in case of the repeated protocol). Control mice received vehicle only. The measurement of nociception severity was determined by the number of abdominal constrictions known as writhing. The number of writhing episodes was recorded over a period of 25 min (Russo et al., 2016).

#### Formalin-Evoked Hind-Paw Licking

Mice received injections of formalin (5% v/v in saline; 20 µl/paw), into the plantar surface of the right hind paw using a 27-gauge needle fitted to a microsyringe. Paw licking was monitored immediately after formalin administration by an observer blind to the experimental treatments during two epochs: 0–15 min (early phase) and 15–45 min (late phase) (Sasso et al., 2012). PER effect was evaluated after single and repeated administrations; formalin test was performed 1 h after the last treatment.

#### Paw Edema

Paw edema was induced by a sub-plantar injection of 50 µl of sterile saline containing 1% λ –carrageenan into the right hind paw. Paw volumes were measured by a plethysmometer apparatus (Ugo Basile, Milan, Italy) at different time intervals: before injection (0) and 1, 3, 5, 24, 48, and 72 h after administration of carrageenan. PER was administered orally at the single dose of 5 mg/kg 30 min before carrageenan injection. The increase of paw volume was evaluated as the difference between the paw volume measured at each time point and the basal paw volume measured immediately before carrageenan injection (D’Agostino et al., 2007).

### Neuropathic Pain model

#### Chronic Constriction Injury

The sciatic nerve of mice was surgically ligated, as previously described (Russo et al., 2016). Briefly, mice (n = 6 each group) were first anesthetized with i.p., injection of xylazine (10 mg/kg) and ketamine (100 mg/kg), and then a small incision in the middle left thigh (2 cm in length) was performed to expose the sciatic nerve. The nerve was loosely ligated at two distinct sites (spaced at a 2-mm interval) around the entire diameter of the nerve using silk sutures (7–0). In sham-operated animals, the nerve was exposed but not ligated. Four days after surgery, mice were treated orally with PER using the dose of 5 mg/kg for acute treatment and 3 mg/kg for repeated administrations, as described above. On day 7 after surgery, mechanical allodynia and hyperalgesia were measured.

#### Mechanical Hyperalgesia

Paw withdrawal threshold (g) to mechanical stimuli was measured using the Randall–Selitto analgesimeter for mice (Ugo Basile, Varese, Italy). Latencies of paw withdrawal to a calibrated pressure were assessed on both ligated and contralateral paws on day before ligation, and again on day 7 following sciatic nerve ligation (CCI), therefore each paw was tested twice per session. Cut-off force was set at 100 g.

#### Mechanical Allodynia

To assess for changes in sensation or in the development of mechanical allodynia, sensitivity to tactile stimulation was measured using the Dynamic Plantar Aesthesiometer (DPA, Ugo Basile, Italy), which is an automated version of the von Frey hair assessment (Mannelli et al., 2013). Mice were placed in Plexiglas boxes (30 × 30 × 25 cm) with a mesh metal floor covered by a plastic dome that enabled the animal to walk freely, but not to jump. When a trial is initiated, the device raises the filament to touch the foot and progressively increases force until the animal withdraws its foot, or until it reaches a maximum of 5 g of force (cut-off). The DPA automatically records the force at which the foot is withdrawn. Mice were acclimated for 15 min to the environment. Each paw was tested twice per session and the test was performed on day before ligation, and again on day 7 following CCI. The means of the paws’ withdrawal was expressed in grams.

### Western Blot Analysis on Spinal Cord

Mice treated with acute (5 mg/kg) or repeated (3 mg/kg) PER doses were euthanized and the spinal cord was removed via dissection for the following determinations. Tissues were homogenized in lysis buffer (50 mM Tris–HCl, pH 7.4; 1 mM EDTA; 100 mM NaCl; 20 mM NaF; 3 mM Na_3_VO_4_; 1 mM PMSF with 1% (v/v) Nonidet P-40; and protease inhibitor cocktail). After 45 min, lysates were centrifuged at 12,000 rpm for 20 min at 4°C and the supernatant was stored at −80°C until use. Protein concentrations were estimated using bovine serum albumin as a standard in a Bradford reagent assay. Proteins were separated on SDS-PAGE, transferred to nitrocellulose membranes, and incubated with the primary anti-CB_1_ antibody (cat no P0369, Sigma Aldrich). The signal was visualized with the ECL system (Pierce) by Image Quant (GE Healtcare, Milan, Italy). Protein bands were densitometrically quantified using Quantity One software (Bio-Rad Laboratories). Membranes were incubated with anti-β-actin (dilution 1:15000; Sigma–Aldrich; Milan Italy) for normalization.

### Real-Time Polymerase Chain Reaction Analysis

Total RNA was extracted from the spinal cord using Trizol (Ambion). Two micrograms of total RNA were used in first-strand cDNA synthesis (Promega, Madison, WI) according to the manufacturer’s instructions. PCRs were performed with a Bio-Rad CFX96 Connect Realtime PCR System instrument and software (Bio-Rad Laboratories). The PCR conditions were 15 min at 95°C followed by 40 cycles of two-step PCR denaturation at 94°C for 15 s, annealing extension at 55°C for 30 s and extension at 72°C for 30 s. Each sample contained 500 ng cDNA in 2X QuantiTech SYBRGreen PCR Master Mix and gene-specific primers for *Tnfa*, *Il1b*, and *Il6* were purchased from Qiagen (Hilden, Germany). The relative expression of mRNA was normalized to *Gapdh* as housekeeping gene, and the data were analyzed according to the 2–ΔΔCT method.

### Statistical Analysis

The results were expressed as mean ± S.E.M. Data were analyzed using t test, one- or two-ways ANOVA (as appropriated) followed by Tukey’s or Bonferroni’s post hoc comparison test. The P-values *p < 0.05, **p < 0.01, and ***p < 0.001 vs. Vehicle group; ^+^ *p* < 0.05, ^++^ *p* < 0.01 vs. CCI-PER; ^#^ *p* < 0.5, ^##^ *p* < 0.01, ^###^ *p* < 0.001, and ^####^ *p* < 0.0001 vs. Vehicle sham group were considered statistically significant.

## Results

### Analgesic Effect of PER on Tail Flick and Hot Plate Latency

**FIGURE 1:**
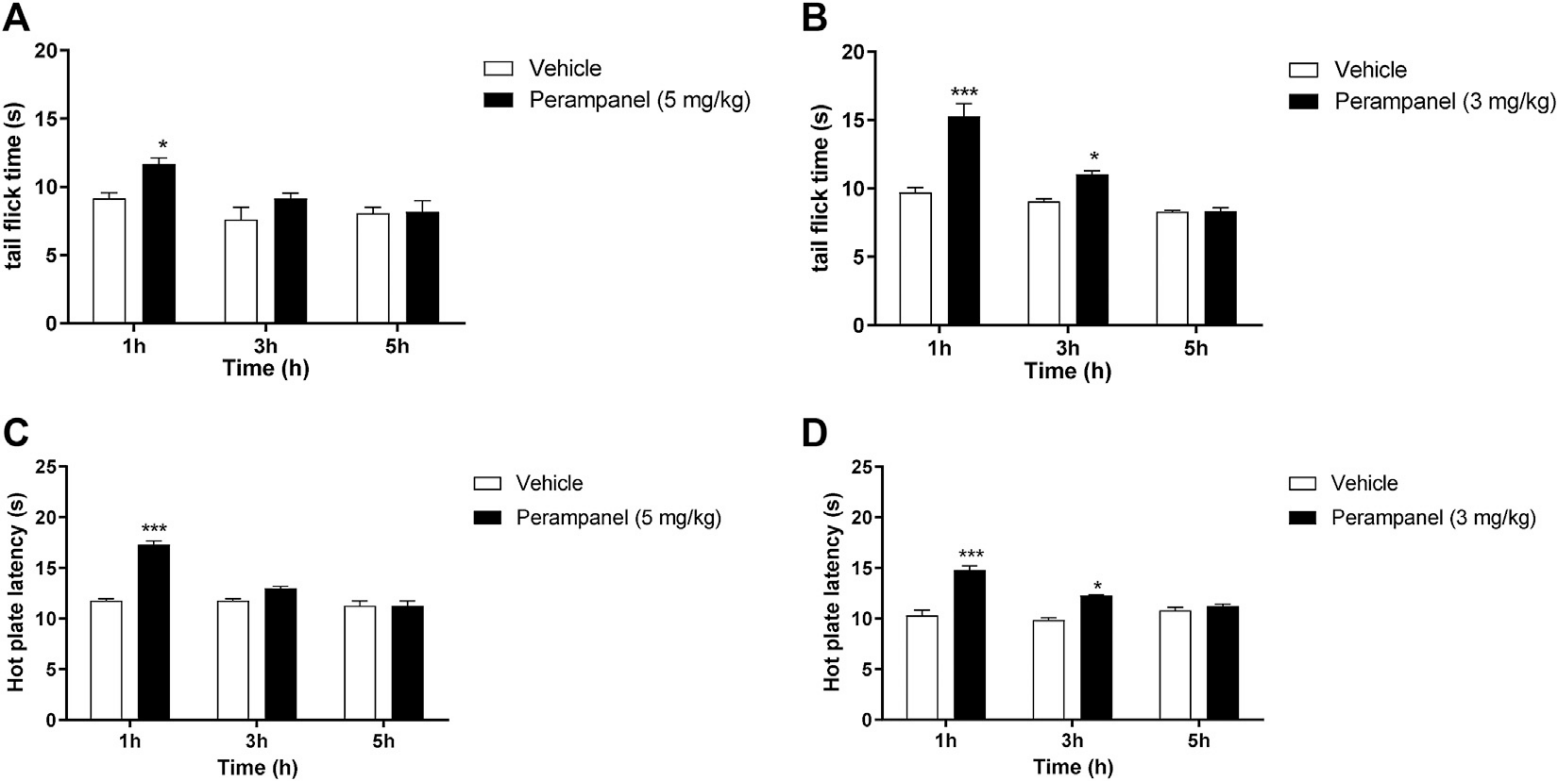
Analgesic effect of PER on tail flick and hot plate tests. Mice were tested 1, 3, and 5 h following acute or repeated oral PER administration. Effect of **(A)** acute PER (5 mg/kg) and **(B)** repeated PER (3 mg/kg/d) for 4 consecutive days administration in the tail flick test. Effect of **(C)** acute PER (5 mg/kg) and **(D)** repeated PER (3 mg/kg/d) for 4 consecutive days in the hot plate test. Data are shown as means ± S.E.M, n = 6–7/group; *p < 0.05, ***p < 0.001 vs. Vehicle, followed by two-way ANOVA Bonferroni’s post-hoc test.

Nociception was firstly evaluated by tail-flick and the hot-plate tests (Figure 1). The tail flick latency, expressed in seconds, was measured at 1, 3, and 5 h after PER administration (Panels A and B). Acute oral administration of PER (5 mg/kg) produced significant changes in tail flick latency only 1 h following administration (*p < 0.05 vs vehicle; F_(2, 30)_ = 17,02 for factor time; F_(1, 30)_ = 10,13 for factor treatment; F_(2, 30)_ = 0,8148 for time X treatment interaction, followed by two-way ANOVA Bonferroni’s post-hoc test)

Figure 1A No effect was observed at 3 and 5 h after the injection. Repeated daily oral administration, using a lower dose of PER (3 mg/kg) for 4 consecutive days, produced a marked and significant increase in the latency of the nociceptive reaction, with a maximal effect 1 h after oral administration (***p < 0.001 vs. vehicle; Figure 1B). This effect remained significant up to 3 h after the last PER administration (*p < 0.05 vs. vehicle; F_(2, 30)_ = 15,79 for factor time; F_(1, 30)_ = 16,79 for factor treatment; F_(2,30)_ = 4,440 for time X treatment interaction, followed by two-way ANOVA Bonferroni’s post-hoc test; Figure 1B).

To confirm these results, we measured heat nociception also using the hot plate test at the same time points. Similarly, single oral administration of PER at the dose of 5 mg/kg significantly attenuated hypersensitivity in response to thermal stimulus during the first hour (***p < 0.001 vs vehicle; F_(2, 36)_ = 25,65 for factor time; F_(1, 36)_ = 15,28 for factor treatment; F_(2, 36)_ = 5,555 for time X treatment interaction, followed by two-way ANOVA Bonferroni’s post-hoc test; Figure 1C), while repeated administrations of PER at the dose of 3 mg/kg significantly reduced hypersensitivity up to 3 h after the last PER administration (***p < 0.001 and *p < 0.5 vs. vehicle; F_(2, 36)_ = 18,11 for factor time; F_(1, 36)_ = 20,43 for factor treatment; F_(2, 36)_ = 3,658 for time X treatment interaction, followed by two-way ANOVA Bonferroni’s post-hoc test; Figure 1D).

### Analgesic Effect of PER on Visceral Pain

**FIGURE 2:**
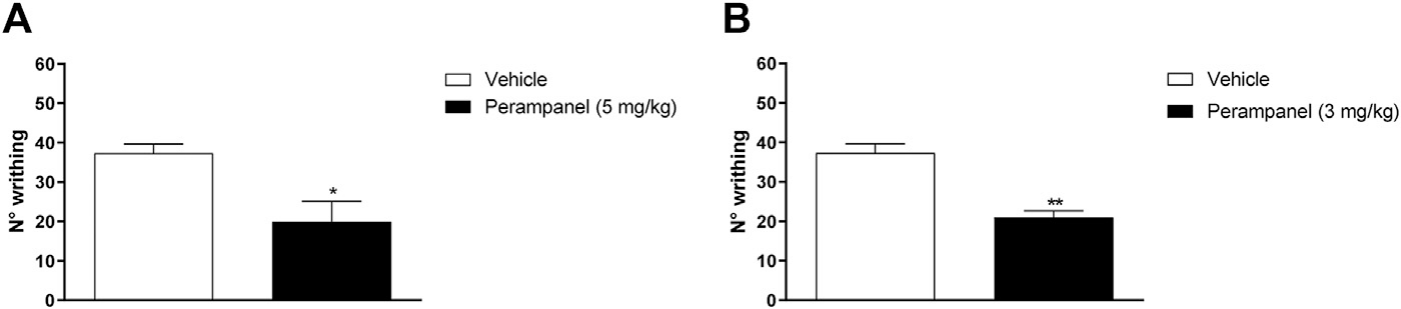
Effect of PER in a model of visceral pain. Mice were tested following the intraperitoneal 0.6% acetic acid injection. **(A)** Number of writhing after a single administration of vehicle or PER (5 mg/kg); **(B)** number of writhing after repeated oral PER (3 mg/kg/d) for 4 consecutive days administration. Data are shown as means ± S.E.M, n = 6–7/group; *p < 0.05, **p < 0.01 PER vs. Vehicle, unpaired t test, two-tailed.

PER efficacy in reducing the nocifensive behavior was studied using the acetic acid-induced writhing test, where i.p., injection of 0.6% acetic acid normally determines approx. 40 writhing episodes (Figures 2A,B). Acute oral administration of PER (5 mg/kg) induced a significant reduction of nocifensive behavior (*p < 0.05 vs. vehicle; t = 3,029 Unpaired t test, two-tailed; Figure 2A). Similarly, repeated oral administration of PER showed significant reduction of writhing episodes compared to controls (**p < 0.001 vs. vehicle; t = 4,269 Unpaired t test, two-tailed; Figure 2B).

### Anti-Inflammatory Effect of PER

**FIGURE 3:**
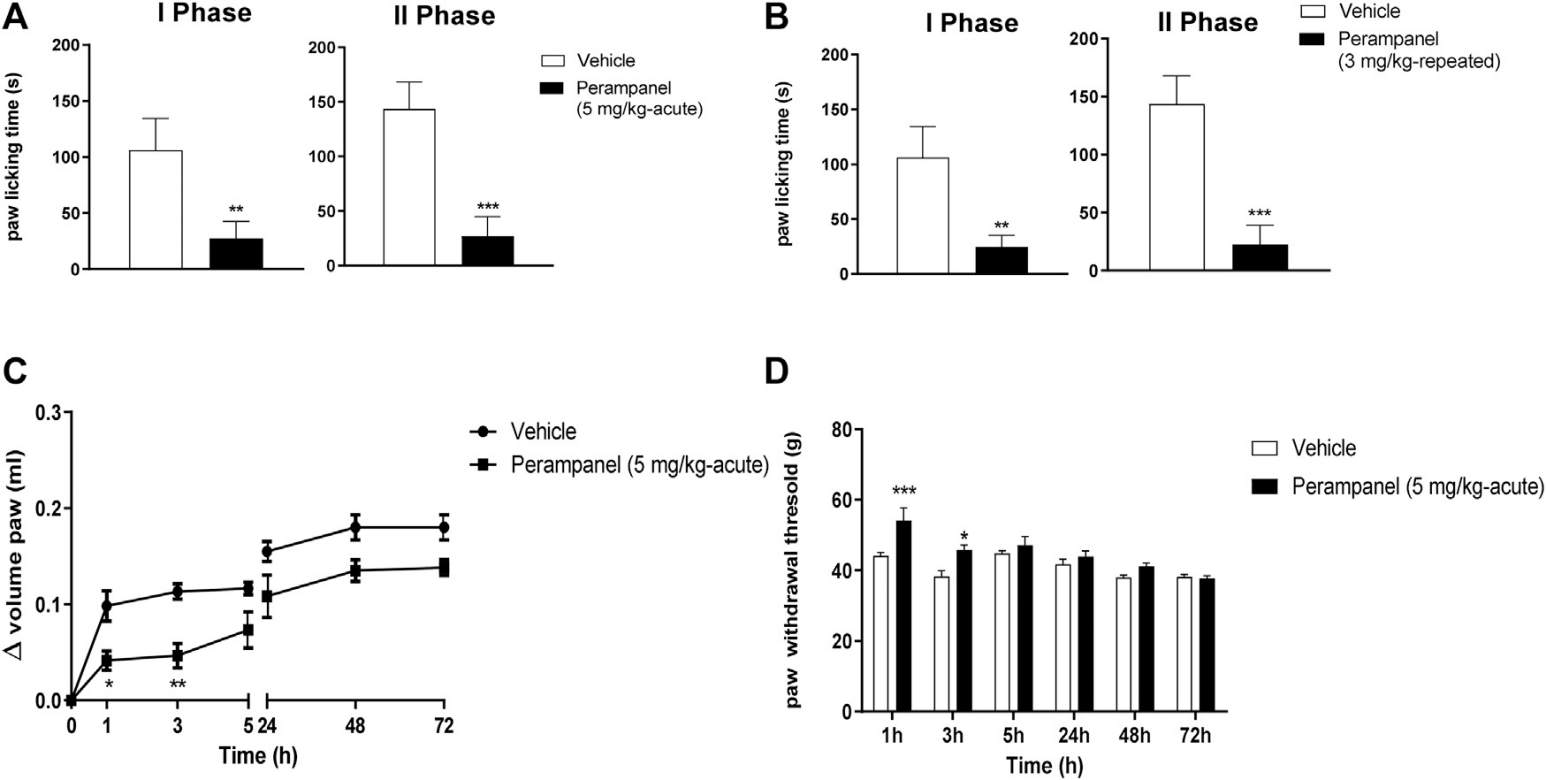
Effect of PER in formalin **(A**,**B)** and carrageenan **(C**,**D)** animal models. In A and B mice were tested following formalin (5%) intraplanar administration; **(A)** time spent in paw licking after a single vehicle or PER (5 mg/kg) administration, **(B)** time spent in paw licking after repeated oral vehicle or PER (3 mg/kg/d) for 4 consecutive days administration. **C** and **D**, mice were tested following carrageenan (1%) intraplanar injection. Paw oedema **(C)** and paw withdrawal latency **(D)** were evaluated following oral acute vehicle or PER (5 mg/kg) administration. Data are shown as means ± S.E.M, n = 6–7/group; *p < 0.05, **p < 0.01, ***p < 0.001 PER vs. Vehicle, followed unpaired t test, two-tailed or two-way ANOVA Bonferroni’s post-hoc test.

The anti-inflammatory effects of PER were tested using the formalin test (Figure 3). Intraplantar administration of formalin induced a marked pain behavior, characterized by two phases, an intense early sensorial phase (0–15 min after injection), and a second inflammatory phase (15–45 min after injection). Single oral administration of PER (5 mg/kg) given 1 h before the test, produced an effect in both phases (**p < 0.001; ***p < 0.0001 vs. vehicle; t = 5.976 Unpaired t test, two-tailed phase I; t = 9.394 Unpaired t test, two-tailed phase II, Figure 3A). A similar effect on both phases was observed also after repeated administrations of PER (3 mg/kg) up to 4 days (**p < 0.001; ***p < 0.0001 vs. vehicle; t = 6.582 Unpaired t test, two-tailed phase I; t = 9.987 Unpaired t test, two-tailed phase II; Figure 3B).

Considering the effect of PER in the second phase of formalin test, known as inflammatory phase, we tested this drug in the carrageenan-induced paw edema model of inflammatory pain (Figures 3C,D). Following carrageenan injection, paw edema develops in two phases: an acute phase in the first 5 h and a second phase peaking at 72 h (28). The acute treatment with PER (5 mg/kg) 30 min before carrageenan administration reduced paw edema in a time-dependent manner. During the first phase (0–5 h), PER inhibited edema formation for 3 h after carrageenan injection (*p < 0.05; **p < 0.01; F_(6, 70)_ = 42,00 for factor time; F_(1, 70)_ = 42,34 for factor treatment; F_(6, 0)_ = 1,435 for time X treatment interaction, followed two-way ANOVA Bonferroni’s post-hoc test; Figure 3C), while in the second phase, PER treatment did to affect edema formation. The same mice were also subjected to the Randall-Selitto test (Figure 3D). Results showed an increased in paw withdrawal latency of PER group in the first 24 h compared to control group (*p < 0.05; ***p < 0.001; F_(5, 60)_ = 12,97 for factor time; F_(1,60)_ = 19,71 for factor treatment; F_(5,60)_ = 2,828 for time X treatment interaction, followed two-way ANOVA Bonferroni’s post-hoc test; Figure 3D), while no effect was observed at 48 and 72 h.

### Involvement of Cannabinergic System on PER’s Anti-Inflammatory Activity

**FIGURE 4:**
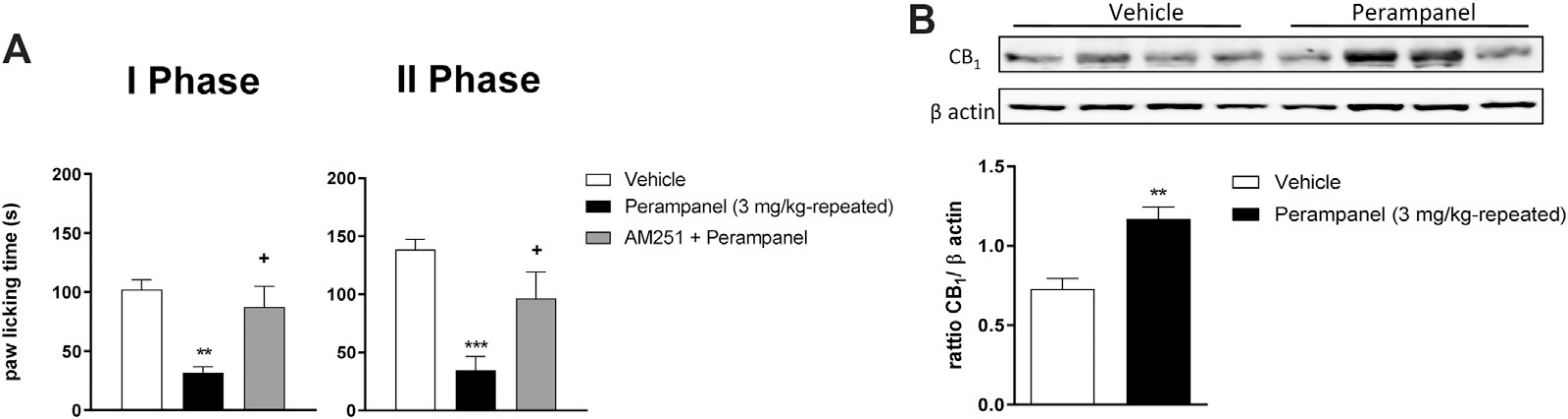
CB1 involvement in PER-induced analgesia in formalin test. **(A)** Time spent in paw licking after a repeated (4 consecutive days) oral vehicle, PER (3 mg/kg) administration. The CB1 antagonist, AM251 (1 mg/kg/ip) was administrated one hour before the last administration. **(B)** CB1 Protein expression in the spinal cord of mice treated with vehicle or PER (3 mg/kg). Data are shown as means ± S.E.M, n = 6–7/group; *p < 0.05, **p < 0.01, ***p < 0.001 vs. Vehicle. +*p* < 0.05 vs. PER followed two-way ANOVA Bonferroni’s post-hoc test or unpaired t test, two-tailed.

The contribution of CB_1_ receptors in PER effects was investigated using the CB1 antagonist AM251 (1 mg/kg/ip), injected one hour before the last PER administration in the repeated scheme of 3 mg/kg. Results clearly showed that AM251 was able to reverse the effect of PER in both phases of formalin test (^**+**^ *p* < 0.05 vs. Perampanel; F_(2, 15)_ = 10,14 for treatment phase I; F_(2, 15)_ = 11,11 for treatment phase II, followed one-way ANOVA Bonferroni’s post-hoc test; Figure 4A). At this experimental time point, CB_1_ protein expression of PER treated mice in the spinal cord was also determined. As reported in Figure 4B western blot analysis, PER treatment induced a significant increase in expression of CB_1_ receptor compared to vehicle group **p < 0.001 vs. vehicle; t = 4,354, Unpaired t test, two-tailed).

### Perampanel Effect in CCI-Induced Neuropathic Pain

**FIGURE 5:**
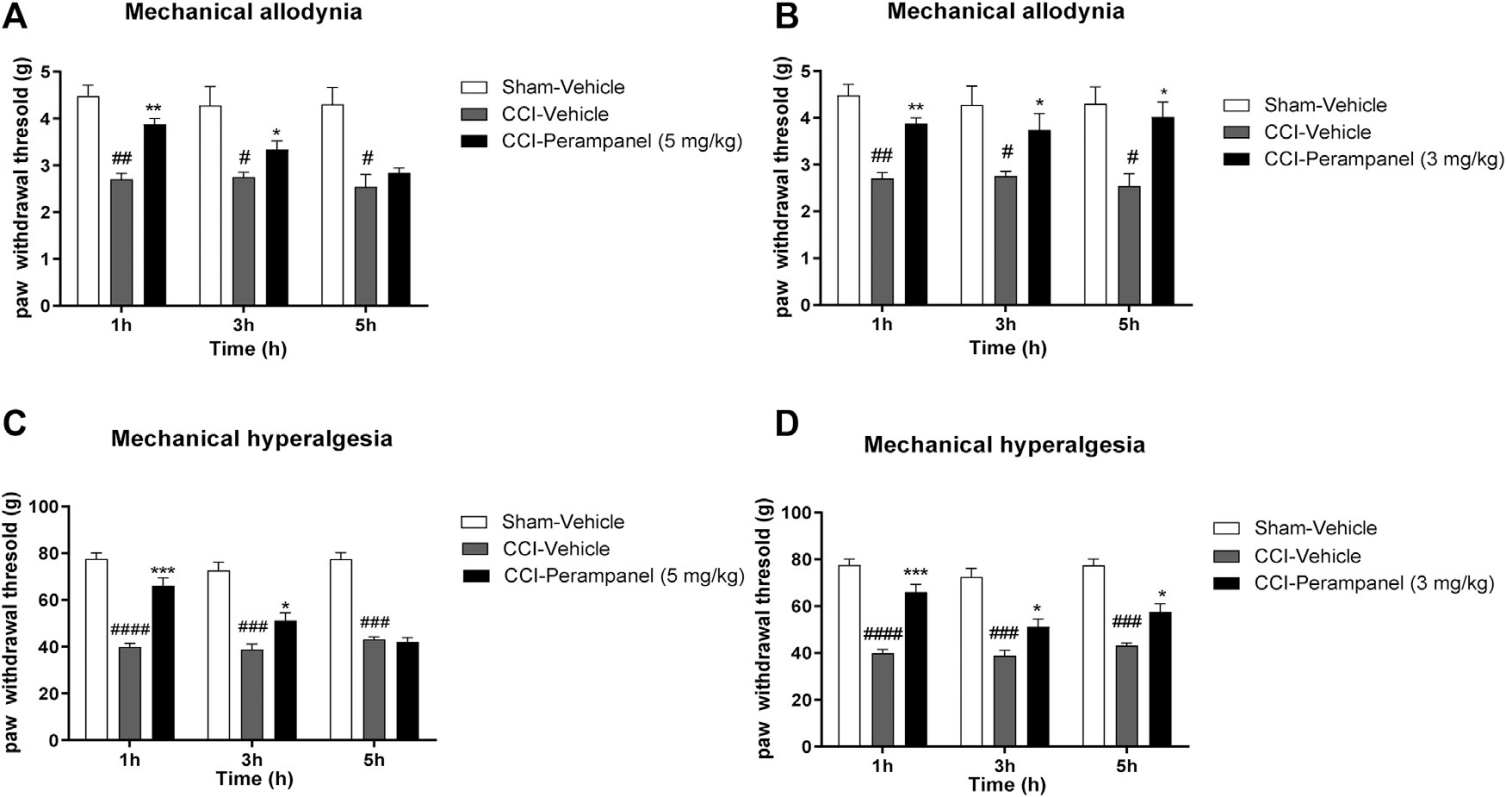
PER effect in CCI-induced neuropathic pain. Time course effect of vehicle or PER after single **(A**,**C)** and repeated for 4 days **(C**,**D)** oral administration. Antiallodynic PER effect after **(A)** acute (5 mg/kg) and **(B)** repeated (3 mg/kg) administration in Von Frey test, on day 7 following sciatic nerve ligation. Antihyperalgesic PER effect after **(C)** acute (5 mg/kg) and **(D)** repeated (3 mg/kg) administration in Randall-Selitto test, on day 7 following sciatic nerve ligation. Sham group represent not ligated animals. Data are shown as means ± S.E.M, n = 6–7/group; *p < 0.05, **p < 0.01, ***p < 0.001 vs. Vehicle. #*p* < 0.05, ##*p* < 0.01, ###*p* < 0.001 vs. Sham-Vehicle, followed by two-way ANOVA Tukey’s post-hoc test.

To examine whether PER was able to reduce allodynia and hyperalgesia associated with NP, we induced peripheral neuropathy in mice by ligation of sciatic nerve. Four days after ligature, we started the acute (5 mg/kg; Figures 5A,C) and repeated (3 mg/kg; Figures 5B,D) treatment with PER given orally. At day 7 we tested pain threshold after thermal and mechanical noxious stimuli. It was observed a significant increase in paw withdrawal threshold of vehicle-treated mice compared to sham mice in response to von-Frey test, suggesting the development of mechanical allodynia and the cute PER administration (5 mg/kg) significantly reduced the number of ipsilateral paw withdrawals. Acute PER administration (5 mg/kg) significantly reduced the number of ipsilateral paw withdrawals at 1 h post treatment (^*#*^ *p* < 0.05; ^##^ *p* < 0.01 vs. Sham; **p < 0.01 vs CCI-vehicle; F_(1,931, 28,96)_ = 1,145 for factor time; F_(2, 15)_ = 29,32 for factor treatment; F_(4, 30)_ = 3,532 for time X treatment interaction, followed by two-way ANOVA Tukey’s post-hoc test; Figure 5A).

Similarly, increased paw withdrawal latency was also observed in Randall-Selitto test, indicating the development of mechanical hyperalgesia until 3 h post treatment (^*###*^ *p* < 0.001, ^####^ *p* < 0.0001 vs. Sham;*p < 0.05; ***p < 0.001 vs. CCI-vehicle; F_(1,783, 26,75)_ = 4,673 for factor time; F_(2, 15)_ = 111,8 for factor treatment; F_(4, 30)_ = 14,93 for time X treatment interaction, followed by two-way ANOVA Tukey’s post-hoc test; Figure 5C).

On the other hand, repeated oral PER administration (3 mg/kg) significantly induced a significant antiallodynic (^*#*^ *p* < 0.05; ^##^ *p* < 0.01 vs. Sham;*p < 0.05; **p < 0.01 vs CCI-vehicle; F_(1,900, 28,49)_ = 0,5204 for factor time; F _(2, 15)_ = 17,63 for factor treatment; F _(4, 30)_ = 1,103 for time X treatment interaction, followed by two-way ANOVA Tukey’s post-hoc test; Figure 5B) and anti-hyperalgesic effect (^*###*^ *p* < 0.001, ^####^ *p* < 0.0001 vs. Sham;*p < 0.05; ***p < 0.001 vs. CCI-vehicle; F_(1,447, 21,70)_ = 4,170 for factor time; F_(2, 15)_ = 121,7 for factor treatment; F_(4, 30)_ = 3,440 for time X treatment interaction, followed by two-way ANOVA Tukey’s post-hoc test; Figure 5D) at all experimental times (1, 3, and 5 h post treatment).

### Involvement of Cannabinergic System on Anti-Neuropathic PER Activity

**FIGURE 6:**
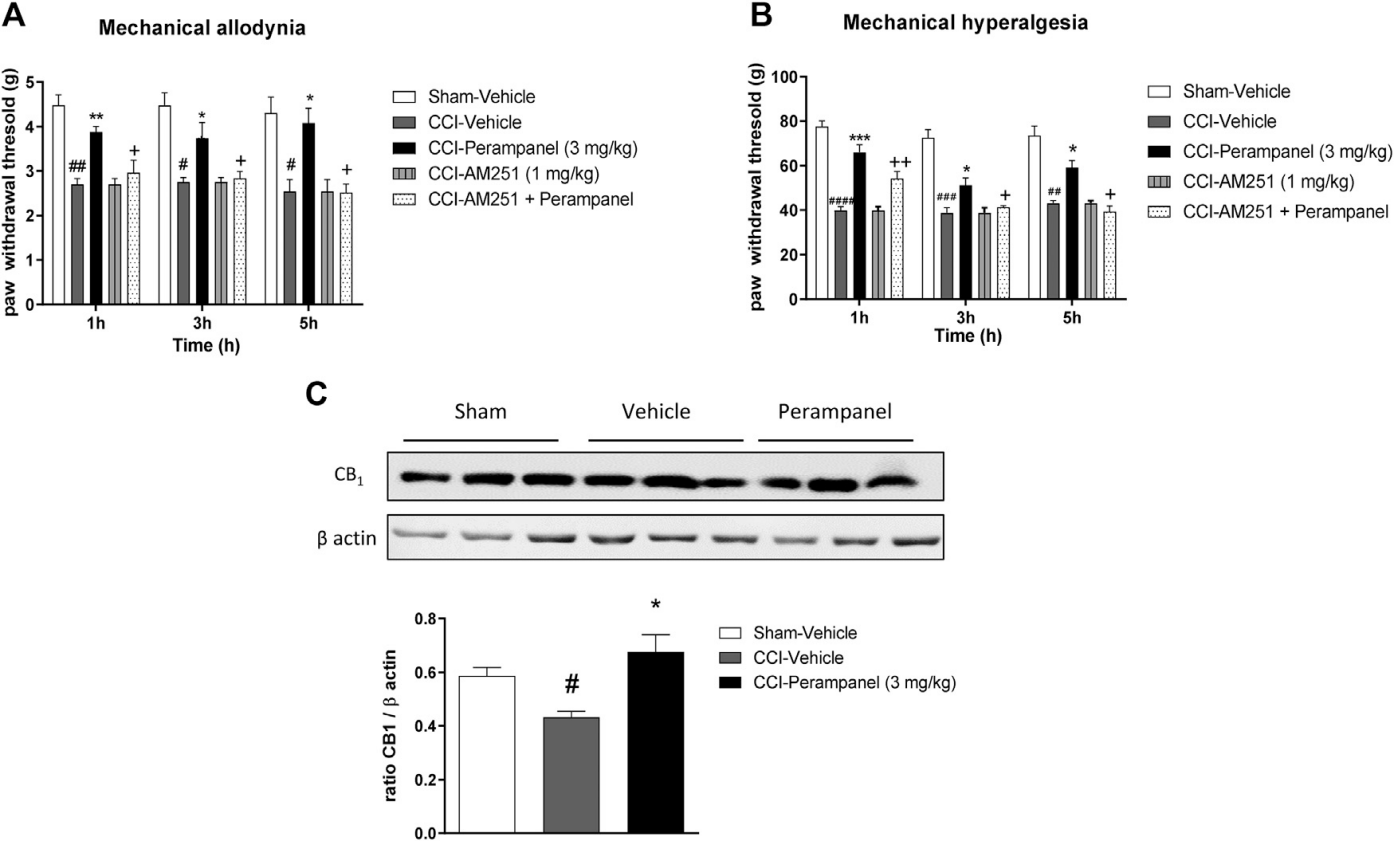
CB1 involvement in PER-induced neuropathic pain reduction. **(A)** CB1 protein expression in the spinal cord of Sham- and CCI-mice treated with vehicle and PER (3 mg/kg). **(B)** Antiallodynic and **(C)** Antihyperalgesic PER effect at 1, 3, and 5 h post treatment with or without CB1 antagonist, AM251, (1 mg/kg/ip). The antagonist was administrated one hour before the last administration. Data are shown as means ± S.E.M, n = 6–7/group; *p < 0.05, **p < 0.01, ***p < 0.001 vs Vehicle. #*p* < 0.05, ##*p* < 0.01, ###*p* < 0.001 vs. Sham-Vehicle. +*p* < 0.05 vs. PER, followed by two-way ANOVA Tukey’s post-hoc test.

Western blot analysis showed that mice with CCI expressed reduced levels of CB1 receptor protein in the spinal cord, while the repeated treatment with PER (3 mg/kg) restored CB1 receptor expression in the spinal cord (^*^ *p* < 0.05 vs. CCI-vehicle; F_(2, 6)_ = 8,158 followed by one-way ANOVA Tukey’s post-hoc test; Figure 6A). These data suggested a potential involvement of the cannabinergic system in the mechanism of action of PER, therefore a specific CB1 antagonist, AM251 (1 mg/kg, ip) was used for the in vivo tests. AM251 was administered 30 min before last PER (3 mg/kg) administration in CCI mice; mice treated with AM251 showed a significant decreased PER effect in both mechanical allodynia and hyperalgesia (^*#*^ *p* < 0.05; ^##^ *p* < 0.01 vs. Sham;*p < 0.05; **p < 0.01 vs. CCI-vehicle; ^+^ *p* < 0.05 vs. CCI-PER; F_(1,809, 45,21)_ = 0,8809 for factor time; F_(4, 25)_ = 30,90 for factor treatment; F_(8, 50)_ = 0,4047 for time X treatment interaction, followed by two-way ANOVA Tukey’s post-hoc test; Figure 6B; (^##^ *p* < 0.01, ^*###*^ *p* < 0.001, ^####^ *p* < 0.0001 vs. Sham; *p < 0.05; ***p < 0.001 vs. CCI-vehicle ^+^ *p* < 0.05 ^++^ *p* < 0.01 vs. CCI-PER; F_(1,977, 49,41)_ = 3,560 for factor time; F_(4, 25)_ = 94,49 for factor treatment; F_(8, 50)_ = 2,173 for time X treatment interaction, followed by two-way ANOVA Tukey’s post-hoc test; Figure 6C).

### Evaluation of Pro-Inflammatory Cytokines in CCI-Mice Spinal Cord

**FIGURE 7:**
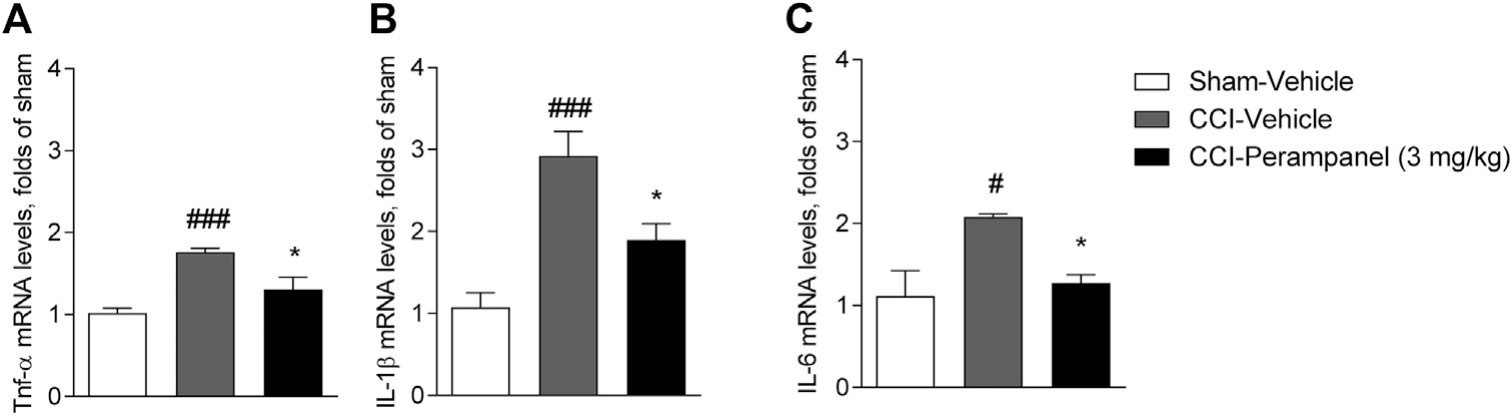
Expression of pro-inflammatory cytokines in spinal cord on day 7 after sciatic nerve ligation. **(A)** mRNA level of TNFα. **(B)** mRNA level of IL-1β. **(C)** mRNA level of IL-6. Data are shown as means ± S.E.M, n = 6–7/group using ordinary one-way ANOVA; *p < 0.05, vs. Vehicle. #*p* < 0.05, ###*p* < 0.001 vs. Sham-Vehicle followed by one-way ANOVA Tukey’s post-hoc test.

Since inflammation has been associated with the pathogenesis and progression of chronic pain, we finally investigated the possible modulation of cytokines involved in the inflammatory process in CCI-mice treated with vehicle and PER at spinal cord level. On day 7 after surgery, CCI vehicle-group showed a significant increased expression of pro-inflammatory cytokines (TNFα, IL-1β, and IL-6) in spinal cord (^#^ *p* < 0.05; ^###^ *p* < 0.0001 vs. sham-Vehicle; Figures 7A–C). Significant reduction of all these cytokines were observed in CCI PER treated mice (*p < 0.05 vs. CCI-vehicle; Figure 7A–C) (TNFα, F_(2,17)_ = 14.21; IL-1β, F_(2,17)_ = 15.23; IL-6, F_(2,17)_ = 6.815; followed by one-way ANOVA Tukey’s post-hoc test).

## Discussion

Pain is a complex debilitating and frustrating pathology that may interfere with sleep, work, and activities, eventually leading to reduced quality of life. At same way also pain management is difficult and can lead to harmful effects if not properly administered and monitored. Numerous types of receptors involved in various signaling pathways are activated in pain perception. They can be considered targets for the modulation of pain and studied to discover novel analgesic molecules. For instance, antagonists of TRPV1, TRPM8, ASICs, AMPA, NMDA, mGlu, NK1, and CGRP receptors have shown to be effective in several animal models of pain (Khan et al., 2019); similarly, the activation of specific pathways, such as opioidergic, serotoninergic and cannabinergic systems has shown analgesic properties by modulating the central and peripheral perception of painful stimuli (Scherrer et al., 2009; Palit et al., 2011; De Gregorio et al., 2019).

In the present, study we evaluated the anti-nociceptive effects of PER, a highly selective non-competitive AMPA receptors antagonist, in several mouse models of acute and chronic pain and for the first time, we assessed a possible correlation between the glutamatergic and the cannabinergic system. PER is currently used in clinics as a broad spectrum ASM but it has shown efficacy also in some models of other neurological disorders such as multiple sclerosis, Parkinson disease and migraine (Akamatsu et al., 2016; Lattanzi et al., 2018; Tringali et al., 2018). Different studies have suggested that in cultured rat cortical neurons, PER inhibits the AMPA-induced increase in intracellular Ca^2+^, while in contrast to its effect on AMPA receptors, PER caused little or no inhibition of NMDA-induced Ca^2+^ responses, indicating a selectivity for AMPA receptors versus NMDA receptors (Hanada et al., 2011). It is well known that AMPA receptors regulate excitatory neurotransmission in pain pathways (Woolf and Salter, 2000). In fact, glutamate release in the spinal dorsal horn after nerve injury and peripheral inflammation is an important contributor to pathological pain (through AMPA receptors) inducing a postsynaptic depolarization that is able to remove the Mg^2+^ block from the NMDA receptor (Ruscheweyh et al., 2011). Moreover, studies in hippocampal slices have also demonstrated that PER selectively blocks AMPA receptor-mediated synaptic transmission and neuronal excitation that are the established causes for the development of NP (Ceolin et al., 2012). It was also shown that spinal AMPA receptors contribute to the central sensitization associated with acute pain. In fact, treatment with different doses of morphine showed hypersensitivity to mechanical stimulation but intrathecal administration of a Ca^2+^ permeable selective AMPA receptor blocker disrupted morphine-induced mechanical sensitivity with an increased GluA4 expression and phosphorylation in homogenates of dorsal horn (Cabañero et al., 2013). Moreover, Tao has also shown that inflammation-induced changes in AMPA receptor subunits (i.e., GluA1 membrane insertion and GluA2 internalization) at postsynaptic membranes of dorsal horn neurons are involved in central sensitization in persistent inflammatory pain (Tao, 2010).

Based on this background, we started to investigate the analgesic effect of PER in the tail flick and hot plate tests in order to evaluate spinal nociceptive reflex and supra-spinally responses, after acute or repeated administrations. We used repeated regimen to understand if a poor-active dose (3 mg/kg) in our experimental condition could to be more efficacy and prolonged in the time, despite its short half-life (1.6 h). In both tests PER showed a significant antinociceptive activity following both acute and chronic regimen. Moreover, animals treated for 4 days showed a more prolonged in time activity but did not correlate to a greater effectiveness between the two posological regimens. Our results confirm that AMPA receptors represent an important target to modulate pain sensation. Then, we better characterized PER effects using acute and chronic animal pain models such as, chemical-induced visceral pain using writhing test, the paw licking test and paw edema to assess somatic pain with inflammatory and neurogenic component, and in pathologic conditions due to neuropathic pain. This approach allowed us to analyze the efficacy of PER oral administration on pain perception and also its capability to modulate inflammation. Previous authors have shown that selective, noncompetitive AMPA receptor antagonists, such as SYM 2206, CFM-2, and GYKI 52466, produced antinociceptive effects in the spinal cord of mice with neuropathic and inflammatory pain (Garry et al., 2003). In agreement with these data, results showed that single (5 mg/kg) and repeated (3 mg/kg/d for 4 days) PER administration significantly attenuated writhing episodes, reduced paw edema and formalin-induced pain. In this last experiment, PER reduced the response in both the early phase in which Aδ and C-fibers are triggered, and the late phase characterized by sensitization of nociceptive spinal neurons (Coderre et al., 1993). This specific effect of PER seems to go beyond the modulation of the AMPA channel, suggesting that probably other systems are involved in its mode of action. A link between the glutamatergic and the cannabinergic systems has been previously demonstrated. The endocannabinoids anandamide (AEA) and 2-arachidonoylglycerol (2-AG) are synthesized “*on demand*” from membrane lipids in response to cellular signals such as activation of the postsynaptic glutamate receptors, moreover, cannabinoid agonists inhibit glutamate release in many synapses in the central nervous system, including the prefrontal cortex (Melis et al., 2004), hippocampus and cerebellum (Misner and Sullivan, 1999), striatum (Huang et al., 2001; Robbe et al., 2001), and spinal cord (Morisset and Urban, 2001). Moreover, Palazzo and coworkers showed that endogenous glutamate could tonically modulate nociception through mGlu and NMDA receptors in the periaqueductal grey (PAG) matter and the stimulation of these receptors seems to be required for cannabinoid-induced analgesia (Palazzo et al., 2001).

In our study, we considered the correlation between AMPA modulation and cannabinergic activity by assessing CB_1_ receptor expression in the spinal cord of mice with CCI and we studied the in vivo efficacy of PER using the CB_1_ receptor antagonist AM251. Results clearly showed that PER effects were cannabinergic system-dependent, in fact AM251 was able to reduce PER activity and western blot analysis showed a significantly increased expression of this receptor in comparison with non-treated animals. It is known that pathological conditions, such as nerve injury and/or peripheral inflammation, are an important contributor to long-term potentiation (LTP) of glutamatergic transmission (Ruscheweyh et al., 2011). Glutamate release via activation of presynaptic AMPA receptors initiates the postsynaptic depolarization necessary to remove the Mg^2+^ block from the NMDA receptor channel, which can sequentially induce Ca^2+^ influx, LTP, and hyperalgesia. In the current study, oral PER administration attenuated mechanical and cold hyperalgesia in CCI mice, in agreement with other authors (Murai et al., 2016; Khangura et al., 2017; Hara et al., 2020). Moreover, CB_1_ antagonist AM251, reverted these effects, underlining once again a strong correlation between PER effect and the cannabinergic activity. Finally, an increasing amount of studies suggest that neuroinflammation and pro-inflammatory cytokines are important factors in the development and maintenance of NP (Sweitzer et al., 2001; Zhang and An, 2007). In our study PER reduced proinflammatory cytokine levels suggesting a reduction of the inflammatory state at spinal cord level.

In conclusion, our evidences support the potential use of PER as an analgesic. By means of specific mouse models of pain, we further extend our knowledge on its mechanism of action indicating a clear involvement of the cannabinergic system as well as an anti-inflammatory action. Therefore, while PER mechanism should be further studied also in order to understand the link between AMPA receptors and cannabinergic systems, its potential clinical use on pain therapy should start to be considered.

## Data Availability Statement

The original contributions presented in the study are included in the article/Supplementary Material, further inquiries can be directed to the corresponding author.

## Ethics Statement

The animal study was reviewed and approved by The Institutional Committee on the Ethics of Animal Experiments (CSV) of the University of Naples “Federico II” and by the Ministero della Salute under protocol no.996/2016-PR.

## Author Contributions

DC, CC, CA, and MC Methodology in vivo, LG data curation, DC, CC, and RR writing—original draft preparation, AG and RE review and editing, AC and DG supervision.

## Funding

This work was partly supported by: 1) the Italian Ministry of Health. Grant No. GR-2013-02355028; 2) Italian Ministry of University and Research (MIUR) Prot. 2017B9NCSX.

## Conflict of Interest

RE has received speaker fees or fundings and has participated in advisory boards for Eisai, Pfizer, GW Pharmaceuticals, UCB pharma, Arvelle Therapeutics.

The remaining authors declare that the research was conducted in the absence of any commercial or financial relationships that could be construed as a potential conflict of interest.

## References

[B1] Adedoyin M. O. Vicini S. Neale J. H. (2010). Endogenous N-acetylaspartylglutamate (NAAG) inhibits synaptic plasticity/transmission in the amygdala in a mouse inflammatory pain model. Mol. Pain. 6, 60 10.1186/1744-8069-6-60 20860833 PMC3152775

[B2] Akamatsu M. Yamashita T. Hirose N. Teramoto S. Kwak S. (2016). The AMPA receptor antagonist perampanel robustly rescues amyotrophic lateral sclerosis (ALS) pathology in sporadic ALS model mice. Sci. Rep. 6, 28649 10.1038/srep28649 27350567 PMC4923865

[B3] Bialer M. (2012). Why are antiepileptic drugs used for nonepileptic conditions? Epilepsia 53 (Suppl. 7), 26–33. 10.1111/j.1528-1167.2012.03712.x 23153207

[B4] Cabañero D. Baker A. Zhou S. Hargett G. L. Irie T. Xia Y. (2013). Pain after discontinuation of Morphine treatment is associated with synaptic increase of GluA4-containing AMPAR in the Dorsal Horn of the Spinal Cord. Neuropsychopharmacology. 38, 1472–1484. 10.1038/NPP.2013.46 23403695 PMC3682142

[B5] Morón L. Bortolotto Z. A. Bannister N. Collingridge G. L. Lodge D. Volianskis A. (2012). A novel anti-epileptic agent, perampanel, selectively inhibits AMPA receptor-mediated synaptic transmission in the hippocampus. Neurochem. Int. 61, 517–522. 10.1016/J.NEUINT.2012.02.035 22433907

[B6] Chaparro L. E. Wiffen P. J. Moore R. A. Gilron I. (2012). Combination pharmacotherapy for the treatment of neuropathic pain in adults. Cochrane Database Syst. Rev. 2012, CD008943 10.1002/14651858.CD008943.pub2 PMC6481651 22786518

[B7] Chizh B. A. Schlütz H. Scheede M. Englberger W. (2000). The N-methyl-D-aspartate antagonistic and opioid components of d-methadone antinociception in the rat spinal cord. Neurosci. Lett. 296, 117–120. 10.1016/s0304-3940(00)01638-4 11108995

[B8] Citraro R. Leo A. Franco V. Marchiselli R. Perucca E. De Sarro G. Russo E. (2017). Perampanel effects in the WAG/Rij rat model of epileptogenesis, absence epilepsy, and comorbid depressive-like behavior. Epilepsia 58, 231–238. 10.1111/epi.13629 27988935

[B9] Russo T. J. Fundytus M. E. McKenna J. E. Dalal S. Melzack R. (1993). The formalin test: a validation of the weighted-scores method of behavioural pain rating. Pain. 54, 43–50. 10.1016/0304-3959(93)90098-A 8378102

[B10] D’Agostino G. La Rana G. Russo R. Sasso O. Iacono A. Esposito E. (2007). Acute intracerebroventricular administration of palmitoylethanolamide, an endogenous peroxisome proliferator-activated receptor-alpha agonist, modulates carrageenan-induced paw edema in mice. J. Pharmacol. Exp. Ther. 322, 1137–1143. 10.1124/jpet.107.123265 17565008

[B11] Calignano E. M. Coggeshall R. E. Carlton S. M. (1997). Peripheral NMDA and non-NMDA glutamate receptors contribute to nociceptive behaviors in the rat formalin test. Neuroreport. 8, 941–946. 10.1097/00001756-199703030-00025 9141069

[B12] De Gregorio D. McLaughlin R. J. Posa L. Ochoa-Sanchez R. Enns J. Lopez-Canul M. (2019). Cannabidiol modulates serotonergic transmission and reverses both allodynia and anxiety-like behavior in a model of neuropathic pain. Pain 160, 136–150. 10.1097/j.pain.0000000000001386 30157131 PMC6319597

[B13] Gobbi V. Luongo L. Guida F. Cristino L. Palazzo E. Russo R. (2012). Effects of intra-ventrolateral periaqueductal grey palmitoylethanolamide on thermoceptive threshold and rostral ventromedial medulla cell activity. Eur. J. Pharmacol. 676, 41–50. 10.1016/j.ejphar.2011.11.034 22178921

[B14] Maione C. Labate A. Maschio M. Meletti S. Russo E. (2017). AMPA receptors and perampanel behind selected epilepsies: current evidence and future perspectives. Expert Opin. Pharmacother. 18, 1751–1764. 10.1080/14656566.2017.1392509 29023170

[B15] Douyard J. Shen L. Huganir R. L. Rubio M. E. (2007). Differential neuronal and glial expression of GluR1 AMPA receptor subunit and the scaffolding proteins SAP97 and 4.1N during rat cerebellar development. J. Comp. Neurol. 502, 141–156. 10.1002/cne.21294 17335044

[B16] Garry E. M. Moss A. Delaney A. O’Neill F. Blakemore J. Bowen J. (2003). Neuropathic sensitization of behavioral reflexes and Spinal NMDA receptor/CaM kinase II interactions are disrupted in PSD-95 mutant mice. Curr. Biol. 13, 321–328. 10.1016/S0960-9822(03)00084-8 12593798

[B17] Gwak Y. S. Kang J. Leem J. W. Hulsebosch C. E. (2007). Spinal AMPA receptor inhibition attenuates mechanical allodynia and neuronal hyperexcitability following spinal cord injury in rats. J. Neurosci. Res. 85, 2352–2359. 10.1002/jnr.21379 17549753

[B18] Hanada T. Hashizume Y. Tokuhara N. Takenaka O. Kohmura N. Ogasawara A. (2011). Perampanel: a novel, orally active, noncompetitive AMPA-receptor antagonist that reduces seizure activity in rodent models of epilepsy. Epilepsia. 52, 1331–1340. 10.1111/j.1528-1167.2011.03109.x 21635236

[B19] Hara K. Haranishi Y. Terada T. (2020). Intrathecally administered perampanel alleviates neuropathic and inflammatory pain in rats. Eur. J. Pharmacol. 872, 172949 10.1016/j.ejphar.2020.172949 31991141

[B20] Huang C. C. Lo S. W. Hsu K. S. (2001). Presynaptic mechanisms underlying cannabinoid inhibition of excitatory synaptic transmission in rat striatal neurons. J. Physiol. (Lond). 532, 731–748. 10.1111/j.1469-7793.2001.0731e.x 11313442 PMC2278571

[B21] Jensen T. S. Finnerup N. B. (2014). Allodynia and hyperalgesia in neuropathic pain: clinical manifestations and mechanisms. Lancet Neurol. 13, 924–935. 10.1016/S1474-4422(14)70102-4 25142459

[B22] Khan A. Khan S. Kim Y. S. (2019). Insight into pain modulation: nociceptors sensitization and therapeutic targets. Curr. Drug Targets 20, 775–788. 10.2174/1389450120666190131114244 30706780

[B23] Khangura R. K. Bali A. Kaur G. Singh N. Jaggi A. S. (2017). Neuropathic pain attenuating effects of perampanel in an experimental model of chronic constriction injury in rats. Biomed. Pharmacother. 94, 557–563. 10.1016/j.biopha.2017.07.137 28780471

[B24] Latremoliere A. Woolf C. J. (2009). Central sensitization: a generator of pain hypersensitivity by central neural plasticity. J. Pain. 10, 895–926. 10.1016/j.jpain.2009.06.012 19712899 PMC2750819

[B25] Lattanzi S. Brigo F. Trinka E. Zaccara G. Cagnetti C. Del Giovane C. (2018). Efficacy and safety of cannabidiol in epilepsy: A systematic review and meta-analysis. Drugs 78, 1791–1804. 10.1007/s40265-018-0992-5 30390221

[B26] Silvestrini A. Giovannini G. Russo E. Meletti S. (2018). The role of AMPA receptors and their antagonists in status epilepticus. Epilepsia. 59, 1098–1108. 10.1111/epi.14082 29663350

[B27] Mannelli L. D. C. D’Agostino G. Pacini A. Russo R. Zanardelli M. Ghelardini C. (2013). Palmitoylethanolamide is a disease-modifying agent in peripheral neuropathy: pain relief and neuroprotection share a PPAR-Alpha-mediated mechanism. Mediators Inflamm. 2013, 328797 10.1155/2013/328797.2013 23533304 PMC3596927

[B28] McRoberts J. A. Coutinho S. V. Marvizón J. C. Grady E. F. Tognetto M. Sengupta J. N. (2001). Role of peripheral N-methyl-D-aspartate (NMDA) receptors in visceral nociception in rats. Gastroenterology 120, 1737–1748. 10.1053/gast.2001.24848 11375955

[B29] Mayer M. Perra S. Muntoni A. L. Pillolla G. Lutz B. Marsicano G. (2004). Prefrontal cortex stimulation induces 2-arachidonoyl-glycerol-mediated suppression of excitation in dopamine neurons. J. Neurosci. 24, 10707–10715. 10.1523/JNEUROSCI.3502-04.2004 15564588 PMC6730123

[B30] Pistis D. L. Sullivan J. M. (1999). Mechanism of cannabinoid effects on long-term potentiation and depression in hippocampal CA1 neurons. J. Neurosci. 19, 6795–6805. 10.1523/JNEUROSCI.19-16-06795.1999 10436037 PMC6782840

[B31] Morisset V. Urban L. (2001). Cannabinoid-induced presynaptic inhibition of glutamatergic EPSCs in substantia gelatinosa neurons of the rat spinal cord. J. Neurophysiol. 86, 40–48. 10.1152/jn.2001.86.1.40 11431486

[B32] Murai N. Sekizawa T. Gotoh T. Watabiki T. Takahashi M. Kakimoto S. (2016). Spontaneous and evoked pain-associated behaviors in a rat model of neuropathic pain respond differently to drugs with different mechanisms of action. Pharmacol. Biochem. Behav. 141, 10–17. 10.1016/J.PBB.2015.11.008 26597514

[B33] Nagakura E. Marabese I. de Novellis V. Oliva P. Rossi F. Berrino L. (2001). Metabotropic and NMDA glutamate receptors participate in the cannabinoid-induced antinociception. Neuropharmacology 40, 319–326. 10.1016/s0028-3908(00)00160-x 11166324

[B34] Maione S. Sheaff R. J. France C. R. McGlone S. T. Potter W. T. Harkness A. R. (2011). Serotonin transporter gene (5-HTTLPR) polymorphisms are associated with emotional modulation of pain but not emotional modulation of spinal nociception. Biol. Psychol. 86, 360–369. 10.1016/j.biopsycho.2011.01.008 21291949

[B35] Rhudy E. Watanabe M. Hartmann B. Grant S. G. Todd A. J. (2008). Expression of AMPA receptor subunits at synapses in laminae I-III of the rodent spinal dorsal horn. Mol. Pain. 4, 5 10.1186/1744-8069-4-5 18215271 PMC2248168

[B36] Potschka H. Trinka E. (2019). Perampanel: does it have broad-spectrum potential?. Epilepsia. 60, 22–36. 10.1111/epi.14456 29953584

[B37] Robbe D. Alonso G. Duchamp F. Bockaert J. Manzoni O. J. (2001). Localization and mechanisms of action of cannabinoid receptors at the glutamatergic synapses of the mouse nucleus accumbens. J. Neurosci. 21, 109–116. 10.1523/JNEUROSCI.21-01-00109.2001 11150326 PMC6762427

[B38] Ruscheweyh R. Wilder-Smith O. Drdla R. Liu X. G. Sandkühler J. (2011). Long-term potentiation in spinal nociceptive pathways as a novel target for pain therapy. Mol. Pain. 7, 20 10.1186/1744-8069-7-20 21443797 PMC3078873

[B39] Russmann V. Salvamoser J. D. Rettenbeck M. L. Komori T. Potschka H. (2016). Synergism of perampanel and zonisamide in the rat amygdala kindling model of temporal lobe epilepsy. Epilepsia. 57, 638–647. 10.1111/epi.13328 26854031

[B40] Russo E. Gitto R. Citraro R. Chimirri A. De Sarro G. (2012). New AMPA antagonists in epilepsy. Exp. Opin. Investig. Drugs. 21, 1371–1389. 10.1517/13543784.2012.705277 22788917

[B41] Russo R. De Caro C. Avagliano C. Cristiano C. La Rana G. Mattace Raso G. (2016). Sodium butyrate and its synthetic amide derivative modulate nociceptive behaviors in mice. Pharmacol. Res. 103, 279–291. 10.1016/j.phrs.2015.11.026 26675718

[B42] Calignano O. Russo R. Vitiello S. Raso G. M. D’Agostino G. Iacono A. (2012). Implication of allopregnanolone in the antinociceptive effect of N-palmitoylethanolamide in acute or persistent pain. Pain. 153, 33–41. 10.1016/j.pain.2011.08.010 21890273

[B43] Calignano G. Imamachi N. Cao Y. Q. Contet C. Mennicken F. O’Donnell D. (2009). Dissociation of the opioid receptor mechanisms that control mechanical and heat pain. Cell. 137, 1148–1159. 10.1016/j.cell.2009.04.019 19524516 PMC3683597

[B44] Basbaum H. S. Sadhotra A. (2016). Current status of the new antiepileptic drugs in chronic pain. Front. Pharmacol. 7, 276 10.3389/FPHAR.2016.00276 27610084 PMC4996999

[B45] Sweitzer S. Martin D. DeLeo J. A. (2001). Intrathecal interleukin-1 receptor antagonist in combination with soluble tumor necrosis factor receptor exhibits an anti-allodynic action in a rat model of neuropathic pain. Neuroscience. 103, 529–539. 10.1016/s0306-4522(00)00574-1 11246166

[B46] Tao Y.-X. (2010). Dorsal horn alpha-amino-3-hydroxy-5-methyl-4-isoxazolepropionic acid receptor trafficking in inflammatory pain. Anesthesiology 112, 1259–1265. 10.1097/ALN.0B013E3181D3E1ED 20395828 PMC2861149

[B47] Tomić M. Pecikoza U. Micov A. Vučković S. Stepanović-Petrović R. (2018). Antiepileptic drugs as analgesics/adjuvants in inflammatory pain: current preclinical evidence. Pharmacol. Ther. 192, 42–64. 10.1016/j.pharmthera.2018.06.002 29909236

[B48] Tringali G. Currò D. Navarra P. (2018). Perampanel inhibits calcitonin gene-related peptide release from rat brainstem in vitro. J. Headache Pain 19, 107 10.1186/s10194-018-0940-5 30419806 PMC6755590

[B49] Woolf C. J. Salter M. W. (2000). Neuronal plasticity: Increasing the gain in pain. Science 288, 1765–1769. 10.1126/science.288.5472.1765 10846153

[B50] Zhang J. M. An J. (2007). Cytokines, inflammation, and pain. Int. Anesthesiol. Clin. 45, 27–37. 10.1097/AIA.0b013e318034194e 17426506 PMC2785020

